# Proinflammatory and Prothrombotic State in Subjects with Different Glucose Tolerance Status before Cardiovascular Disease

**DOI:** 10.1155/2014/631902

**Published:** 2014-03-17

**Authors:** Irma Isordia-Salas, María Eugenia Galván-Plata, Alfredo Leaños-Miranda, Eberth Aguilar-Sosa, Francisco Anaya-Gómez, Abraham Majluf-Cruz, David Santiago-Germán

**Affiliations:** ^1^Unidad de Investigación Médica en Trombosis, Hemostasia y Aterogénesis, H.G.R. No. 1 “Dr. Carlos Mac Gregor Sánchez Navarro” Instituto Mexicano del Seguro Social, Apartado Postal B 32, Coahuila No. 5, 06703 México, DF, Mexico; ^2^Servicio de Medicina Interna, UMAE, Hospital de Especialidades, Centro Médico Nacional Siglo XXI, Instituto Mexicano del Seguro Social, 06720 México, DF, Mexico; ^3^Unidad de Investigación Médica en Medicina Reproductiva, UMAE H.G.O. No. 4, Instituto Mexicano del Seguro Social, 01070 México, DF, Mexico; ^4^Servicio de Medicina Interna, H.G.Z. A 2 “Francisco del Paso y Troncoso”, del Instituto Mexicano del Seguro Social, 08400 México, DF, Mexico; ^5^Servicio de Medicina Interna, H.G.R. No. 1 “Dr. Carlos Mac Gregor Sánchez Navarro” Instituto Mexicano del Seguro Social, 03100 México, DF, Mexico; ^6^Servicio de Urgencias, H.G.R. No. 1 “Dr. Carlos Mac Gregor Sánchez Navarro” Instituto Mexicano del Seguro Social, 03100 México, DF, Mexico

## Abstract

*Background*. Inflammation has been associated with insulin resistance, type 2 diabetes mellitus (T2DM), and atherothrombosis. *Aim*. To determine differences in levels of proinflammatory and prothrombotic markers such as high sensitivity C-reactive protein (hs-CRP) and fibrinogen in subjects with normal glucose tolerance (NGT), prediabetes, and T2DM and to establish their relationship with other cardiovascular risk factors before clinical manifestations of cardiovascular disease. *Methods*. We conducted a nonrandomized, cross-sectional assay in a hospital at México City. The levels of hs-CRP and fibrinogen were measured and compared according to glucose tolerance status. *Results*. We enrolled 1047 individuals and they were distributed into NGT *n* = 473, pre-DM *n* = 250, and T2DM *n* = 216. There was a statistical difference between NGT and T2DM groups for fibrinogen (*P* = 0.01) and hs-CRP (*P* = 0.05). Fibrinogen and hs-CRP showed a significant positive correlation coefficient (*r* = 0.53, *P*<0.0001). In a multiple stepwise regression analysis, the variability in fibrinogen levels was explained by age, HbA1c, and hs-CRP (adjusted *R* ^2^ = 0.31, *P*<0.0001), and for hs-CRP it was explained by BMI and fibrinogen (adjusted *R* ^2^ = 0.33, *P*<0.0001). *Conclusion*. Inflammation and prothrombotic state are present in people with T2DM lacking cardiovascular disease. Fibrinogen and Hs-CRP are positively correlated. Fibrinogen and hs-CRP concentrations are predominantly determined by BMI rather than glucose levels.

## 1. Introduction

Cardiovascular disease (CVD) is the first cause of death all over the world, accounting for 17.3 million deaths per year, a number that is expected to grow more than 23.6 million by 2030 [1]. Atherosclerosis is the commonest cause of CVD. Currently, atherosclerosis is considered an inflammatory disease triggered by cholesterol-rich lipoproteins and other noxious factors such as cigarette smoke, diabetes mellitus, and hypertension [2]. Increasing evidence supports the concept that atherosclerosis is initiated through developmental in utero processes beginning before birth [3]. There is a long time delay between the start of atherosclerosis and the first clinical manifestation many decades later, supporting the hypothesis that subclinical atherosclerosis represents the vessel memory of lifetime risk factor exposure [4]. In addition, some studies have shown a correlation between subclinical atherosclerosis and selected proinflammatory factors such as C-reactive protein (CRP), fibrinogen, tumor necrosis factor-*α* (TNF-*α*), and interleukin-6 (IL-6) [5]. Furthermore, these selected inflammatory markers seem to be associated with destabilization of atherosclerotic plaque, rather than degree of artery stenosis [6]. Both fibrinogen and CRP are acute-phase reactants produced by the liver. Fibrinogen is a precursor of fibrin, a protein essential for blood clotting and one of the main factors determining blood viscosity and platelet aggregation [7], and elevated levels of CRP have been associated with an elevated risk of adverse ischaemic events and hypercoagulability [8].

The increased prevalence of CVD morbidity and mortality is being followed by an increasing prevalence of diabetes [9]. Worldwide estimation by 2030 predicts more than 470 and 439 million adults with prediabetes (pre-DM) and diabetes mellitus, respectively [10, 11]. Pre-DM is the intermediate stage between normal glucose tolerance (NGT) and diabetic glucose thresholds and occurs 5 to 10 years before the diagnosis of type 2 diabetes mellitus (T2DM) [12, 13]. Even though all individuals with T2DM passed through a pre-DM phase, not all individuals with pre-DM will become T2DM [14]. Approximately, 5 to 10% of people per year with pre-DM will progress to T2DM, with the same proportion converting back to NGT [10]. Two different types of pre-DM have been described: those individuals with hepatic insulin resistance and impaired suppression of hepatic glucose production manifested by hyperglycemia at fast and those individuals with muscle insulin resistance and impaired glucose uptake manifested by postprandial hyperglycemia; both conditions are known as impaired fasting glucose (IFG) and impaired glucose tolerance (IGT), respectively [12]. There is evidence to support an inflammatory contribution for the development of pre-DM and T2DM. Increased levels of high sensitivity C-reactive protein (hs-CRP) and plasminogen activator inhibitor type-1 (PAI-1) are associated with insulin resistance rather than impaired pancreatic *β*-cell function in prediabetic individuals [15]. Also, hs-CRP and PAI-1 have shown to be predictors of incipient diabetes [16].

Although the risk of CVD already exists before the onset of diabetes mellitus since other cardiovascular risk factors are still prevalent in prediabetic subjects, a gradual increment of independent risk of CVD has been reported as blood glucose levels up even under prediabetic glucose thresholds [17]. However, whether IFG or IGT differs in the magnitude of the risk of CVD is still not well established yet [18]. How glucose tolerance status affects the inflammatory and thrombotic response before the development of clinical manifestations of CVD is poorly understood.

Because inflammation is a common feature between atherosclerosis, insulin resistance, and T2DM, and atherothrombosis might exhibit many years before the onset of T2DM, the objective of the present study was to evaluate serum levels of some proinflammatory and prothrombotic markers such as hs-CRP and fibrinogen in subjects with NGT, pre-DM, and T2DM, and to establish their relationship with other traditional cardiovascular risk factors before clinical manifestations of CVD.

## 2. Materials and Methods

We conducted an observational, analytic, and nonrandomized cross-sectional assay in a secondary care level hospital at México City from May 2010 to May 2011. We screened apparently healthy members of the medical staff and relatives of outpatients who came to medical consultation, as well as those who were in routine follow-up for diabetes mellitus. The recruitment was made by invitation through printed announcements and personal appeal to people to participate in the survey if they were interested to know their glucose tolerance status and cardiovascular risk factors. We included all individuals ≥20 years old who accepted to participate. Informed written consent was obtained from all subjects before enrollment. The study protocol was reviewed and approved by the Human Ethical Committee and Medical Research Council of the Instituto Mexicano del Seguro Social and conforms to the ethical guidelines of the 1975 Declaration of Helsinki.

The exclusion criteria were subjects with previous or current diagnosis of CVD (i.e., myocardial infarction, angina, stroke, transient ischemic attack, and peripheral artery disease), cancer, autoimmune disorders, acute and chronic infectious diseases, and hepatic or renal failure and those under immunosuppressive therapy and transplant receivers.

Demographic and clinical data, anthropometric measures, and a fasting blood sample were taken from each subject. The same physician interviewed all the participants in private using a questionnaire and collected the following information: sex, age, smoking status, previous diseases, and familial history of diabetes and CVD. The subjects were considered smokers if they were currently smoking or stopped smoking at least one year before the examination. A positive familial history of diabetes was defined as parents or siblings having T2DM. A familial history of CVD disease was defined as acute myocardial infarction, stroke, or sudden death in a first-degree male relative younger than 55 years of age or a female relative younger than 65 years of age. Same physician performed the anthropometric measurements. Waist circumference (WC) was measured at the midpoint between the last rib and the iliac crest. Body weight was measured by precision scale while subjects were minimally clothed without shoes. Height was measured in a standing position without shoes using measuring tape mater while the shoulders were in a normal state. Body mass index (BMI) was calculated as weight in kilograms divided by height in meters squared. Patients were defined as overweight with a BMI of 25.0–29.9 kg/m^2^ and obese with a BMI ≥30 kg/m^2^. Hypertension was defined as a mean systolic blood pressure of ≥140 mmHg or a mean diastolic blood pressure of ≥90 mmHg resulted from two separate measures at rest, or previous diagnosis.

After an overnight fast of at least eight hours, venous blood was sampled for measurement of serum glucose, glycosylated hemoglobin (HbA1c), hs-CRP, fibrinogen, triglycerides, and high-density lipoprotein cholesterol (HDL-C). The hs-CRP was measured with a high-sensitivity assay with an interassay coefficient of variation (CV) of 8.9% [19]. Fibrinogen was measured in citrated plasma with a modified clot-rate assay using the Diagnostica STAGO ST4 instrument [20] and based on the original method of Clauss [21] with a CV of 3.0%.

The overall sample was categorized according to glucose tolerance status assessed by fasting plasma glucose (FPG) and HbA1c levels as [22]: (1) group with NGT defined as a FPG <100 mg/dL and HbA1c <5.7%; (2) group with Pre-DM defined as a FPG concentration of 100 to 125 mg/dL or HbA1c of 5.7 to 6.4%; and (3) group with T2DM defined as a FPG ≥126 mg/dL or previous diagnosis. The pre-DM group was stratified as follows: (1) subjects with impaired fasting glucose (IFG) defined as a FPG of 100–125 mg/dL plus HbA1c <5.7% and (2) subjects with impaired glucose tolerance (IGT) defined as a FPG <100 mg/dL plus HbA1c 5.7 to 6.4%.

*Statistical Analysis*. The overall sample was stratified according to glucose tolerance status. Demographic and clinical data between subjects with NGT, pre-DM, and T2DM were compared using one-way analysis of variance (ANOVA) for continuous variables, whereas the chi-squared test (*χ* ^2^) was used to compare the categorical parameters. Continuous data were expressed as mean ± standard deviation (SD) and median (interquartile range). Categorical data were expressed as total number plus percentage. A Pearson correlation analysis was performed to the overall sample by standard methods to evaluate the association between proinflammatory and prothrombotic markers with traditional cardiovascular risk factors. A multiple linear stepwise regression analysis was performed to develop a model including all the variables significantly associated with hs-CRP and fibrinogen in the correlation analysis as independent variables to estimate contribution of each independent feature to variation in hs-CRP and fibrinogen levels. Data analysis was performed after natural logarithmic transformation. A *P* value ≤0.05 (two tailed) was considered as statistically significant. All statistical analyses were performed using SPSS (Statistical Package for the Social Sciences) statistical software package (version 15: SPSS Inc., Chicago, IL, USA).

## 3. Results

**Figure 1:**
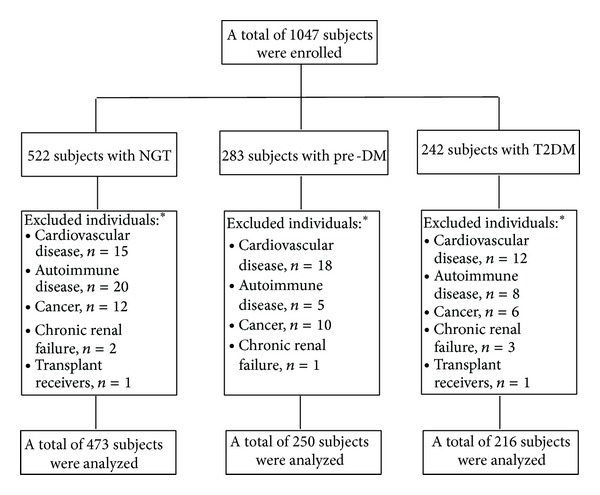
Enrollment. * Some individuals had two or more conditions.

**Figure 2:**
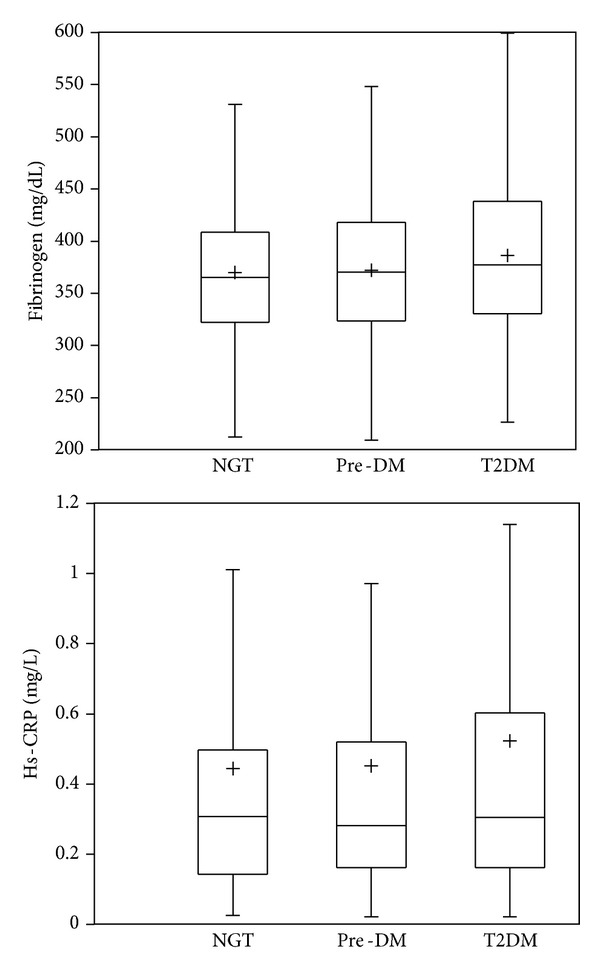
Box plots showing plasma levels of fibrinogen and hs-CRP according to glucose tolerance status. Fibrinogen showed significant difference between NGT and T2DM (*P* = 0.01), but it was not between NGT and pre-DM (*P* = 0.71). The hs-CRP concentration was statistically different between NGT and T2DM (*P* = 0.05), but it was not between NGT and preDM (*P* = 0.89). + = mean.

A total of 1047 individuals were enrolled, Figure 1. One hundred and eight subjects were excluded from the analysis because the presence of exclusion criteria; some of them had two or more conditions: cardiovascular disease (*n* = 45), autoimmune disorders (*n* = 33), cancer (*n* = 28), renal failure (*n* = 6), and transplant receivers (*n* = 2). Demographic and clinical characteristics of 939 subjects are present in Table 1. Individuals were distributed according to glucose tolerance status into three groups: NGT 50.4% (*n* = 473), pre-DM 26.6% (*n* = 250), and T2DM 23% (*n* = 216). There were no differences regardless of gender, smoking status, and familial history of CVD between all groups. However, age, BMI, and WC showed a statistical difference between NGT and pre-DM, pre-DM and T2DM, and NGT versus T2DM groups, with an older age and higher values of abdominal perimeter and BMI in the diabetes mellitus group. Hypertension cases were more frequent in subjects with T2DM compared with pre-DM and NGT, but cases with familial history of T2DM were higher only in diabetic subjects when compared with NGT.  Table 2 shows the biochemical characteristics of 939 subjects according to glucose tolerance status. There was a statistically significant difference of FPG and HbA1c plasma levels between the three groups with a progressive increase from NGT to pre-DM group and a highest value in the T2DM group. Plasma triglyceride levels were higher in T2DM and pre-DM groups when contrasted with NGT, and HDL-C concentrations were significantly lower in T2DM group compared with NGT.  Figure 2 shows the plasma levels of fibrinogen and hs-CRP according to glucose tolerance status. There was a significant difference between NGT and T2DM groups for fibrinogen (369.8 ± 69.1 versus 385.9 ± 77.8, *P* = 0.01) and between NGT and T2DM for hs-CRP (0.4 ± 0.5 versus 0.5 ± 0.6, *P* = 0.05). But, there was not a statistical difference between NGT and pre-DM groups for fibrinogen (369.8 ± 69.1 versus 372 ± 69.8, *P* = 0.71) as well as for hs-CRP (0.4 ± 0.5 versus 0.4 ± 0.7, *P* = 0.89) concentrations, or between pre-DM and T2DM subjects. In a subgroup analysis, when we stratified pre-DM cases assessed by FPG and HbA1c, 95.2% (*n* = 238) accomplished with IFG criteria and 2.8% (*n* = 7) with IGT, and 2% (*n* = 5) shared an IFG plus IGT state (data not shown).

Table 3 shows a correlation analysis among proinflammatory and prothrombotic markers with traditional cardiovascular risk factors of the overall sample. Fibrinogen showed a significant positive correlation coefficient with BMI (*r* = 0.25, *P* < 0.0001), WC (*r* = 0.18, *P* = 0.000), age (*r* = 0.14, *P* = 0.005), HbA1c (*r* = 0.14, *P* = 0.004), and FPG (*r* = 0.10, *P* = 0.03). In turn hs-CRP was positively associated with BMI (*r* = 0.33, *P* < 0.0001), WC (*r* = 0.27, *P* < 0.0001), FPG (*r* = 0.14, *P* = 0.004), and HbA1c (*r* = 0.12, *P* = 0.013). Both proinflammatory (hs-CRP) and prothrombotic (fibrinogen) markers were moderately correlated (*r* = 0.53, *P* < 0.0001). Glucose and HbA1c (*r* = 0.77, *P* < 0.0001) as well as BMI and WC (*r* = 0.82, *P* < 0.0001) showed the strongest correlation coefficients (data not shown). A multiple linear stepwise regression analysis for fibrinogen and hs-CRP as dependent variables is shown in Table 4. The variability of serum levels of fibrinogen was explained only in 31% by a model including age, BMI, WC, FPG, HbA1c, and hs-CRP (adjusted *R* ^2^ = 0.31, *P* < 0.0001). The significant determinants of serum fibrinogen levels were age (*β* = 0.14, 95% CI [0.06–0.23], *P* = 0.001), HbA1c (*β* = 0.12, 95% CI [–0.003–0.25], *P* = 0.05), and hs-CRP (*β* = 0.51, 95% CI [0.42–0.60], *P* < 0.0001). The variability of serum levels of hs-CRP was explained only in 33% by a model including BMI, FPG, HbA1c, and fibrinogen (adjusted *R* ^2^ = 0.33, *P* < 0.0001). The significant determinants of serum hs-CRP levels were BMI (*β* = 0.19, 95% CI [0.04–0.34], and *P* = 0.01) and fibrinogen (*β* = 0.48, 95% CI [0.39–0.56], *P* < 0.0001).

## 4. Discussion

According to our results, subjects with T2DM without obvious cardiovascular complications showed statistically significant higher concentrations of fibrinogen (*P* = 0.01) and hs-CRP (*P* = 0.05) compared with apparently healthy individuals with NGT. However, subjects with pre-DM assessed by FPG and HbA1c tests, whose 95.2% coursed with IFG, had slightly increased concentrations of fibrinogen (*P* = 0.71) and hs-CRP (*P* = 0.89) compared with individuals with NGT without reaching statistical significance. Similar to our results, in the Third National Health and Nutrition Examination Survey (NHANES-III) participants with IFG, newly diagnosed diabetes and previously diagnosed diabetes had 0.9 (0.72–1.37), 1.84 (1.25–2.71), and 1.59 (1.25–2.01) odds of having an elevated CRP concentration after adjustment for age, sex, ethnicity, and BMI [23, 24]. Lucas-Luciardi et al. in a case-control study founded significant increased levels of fibrinogen, PAI-1, and D-dimer, but did not for hs-CRP, in a small sample of subjects with IGT assessed by oral glucose tolerance test (OGTT) without documented CVD [25]. In addition, Gui et al. reported a gradual increase of hs-CRP levels in patients with angiographically documented coronary artery disease as glucose tolerance status changes from NGT to IGT to T2DM, but only with a statistical difference between subjects with NGT and T2DM [26]. In turn, Xiang et al. founded higher levels of hs-CRP in T2DM versus NGT in Chinese population [27]. In contrast, The Insulin Resistance Atherosclerosis Study (IRAS) reported an independent negative association between hs-CRP and insulin sensitivity in healthy nondiabetic subjects [28]. Festa et al. showed higher levels of hs-CRP and PAI-1 in individuals with increased insulin resistance who converted to T2DM in a mean period of 5.2 years versus converters with decreased first-phase insulin secretion, suggesting that insulin resistance rather than impaired *β*-cell function contributes to the proinflammatory state in prediabetic individuals [15]. Evidence suggests that a proinflammatory state is associated with insulin resistance, and an inflammatory profile of people with pre-DM might help to differentiate those individuals with an increased risk to develop T2DM.

In our study, despite the increased levels of fibrinogen and hs-CRP observed in subjects with pre-DM and T2DM when compared to subjects with NGT, the mean thresholds of fibrinogen and hs-CRP into all three groups were inside ranges considered as normal (200–400 mg/dL and <3 mg/L, resp.) [29]. Recently, Samaropoulos et al. reported an association between intensive glycemic control and a reduction in hs-CRP levels in subjects with T2DM [30]. Also, Bembde et al. observed a positive correlation between HbA1c and fibrinogen (*r* = 0.49) in diabetic patients assuming that the poorer the glycemic status, the higher the fibrinogen levels [31]. The apparently correct glycemic control according to mean levels of FPG (158 ± 68.1 mg/dL) and HbA1c (5.9 ± 2.1%) showed in the T2DM group might explain, at least partially, the mean normal ranges of fibrinogen and hs-CRP observed in our sample. Although we did not compare fibrinogen and hs-CRP levels in diabetic subjects with versus without an optimal glycemic control, we founded the highest plasma levels of proinflammatory and prothrombotic markers in subjects with T2DM. The use of different medications such as statins, fibrates, salicylic acid, and thiazolidinediones reduce serum hs-CRP levels. The Multi-Ethnic Study of Atherosclerosis (MESA) showed lower plasma levels of hs-CRP in subjects free of clinical CVD who were using statins than nonusers but did not for fibrinogen and PAI-1 concentrations [32]. According to our results, at the time of the blood sample we founded 54.9% (*n* = 260) subjects with dyslipidemia in the group of NGT, 65.2% (*n* = 163) in the group with pre-DM, and 65.7% (*n* = 142) in the group with T2DM. Each group was receiving medical treatment with statins or fibrates in only 13.9% (*n* = 66), 19.6% (*n* = 49), and 35.6% (*n* = 77) of cases for NGT, pre-DM, and T2DM, respectively. It will be interesting to determine the effect of glycemic control and medication employed in the proinflammatory and prothrombotic markers in future studies.

Another finding in our study was a positive correlation between hs-CRP and fibrinogen of *r* = 0.53 (*P* < 0.0001). This might suggest a crosstalk between coagulation and inflammation, by which an inflammatory response shifts the hemostatic system toward a prothrombotic state [33]. In summary, there are three mechanisms by which inflammation regulates coagulation: (1) inflammatory mediators initiate the coagulation system by promotion of tissue factor expression on the cell surface of monocytes, elevating the platelet count and platelet reactivity, and enhancing the expression of adhesion molecules, reactive oxygen species (ROS), and fibrinogen; (2) inflammation decreases the activity of natural anticoagulant mechanisms, such as tissue factor pathway inhibitor (TFPI), the heparin-antithrombin pathway, and the protein C anticoagulant pathway; and (3) inflammation impairs the fibrinolytic system promoting the formation of PAI-1 [34, 35].

On the other hand, we observed a variability of plasma fibrinogen concentrations of 31% (adjusted *R* ^2^ = 0.31, *P* < 0.0001) determined by hs-CRP (*β* = 0.51 [0.42–0.60], *P* < 0.0001), age (*β* = 0.14 [0.06–0.23], *P* = 0.001), and HbA1c (*β* = 0.12 [–0.003–0.25], *P* = 0.05). Fibrinogen is determined by several modifiable and nonmodifiable factors like age, gender, smoking, BMI, glycemic control, and lipid profile [31]. Also, estimates of the heritability of plasma fibrinogen concentrations range from 34% to 50% [36]. Interestingly, fibrinogen was predominantly determined by hs-CRP in our sample, which is considered an acute phase reactant that promotes an increase of fibrinogen concentrations [34]. In turn, T2DM alters fibrinogen and fibrin structure by glycation diminishing the susceptibility to degradation and promotes their accumulation [37]. Regardless of hs-CRP, we reported a variability of 33% (adjusted *R* ^2^ = 0.33, *P* < 0.0001) determined by fibrinogen (*β* = 0.48 [0.39–0.56], *P* < 0.0001) and BMI (*β* = 0.19 [0.04–0.34], *P* = 0.01). A preceding study in Mexican population showed higher mean serum hs-CRP levels above 5 mg/L in obese subjects and obese plus T2DM individuals, compared with a mean hs-CRP levels of 1.8 (0.9–3.9) mg/L in diabetic individuals without obesity [38]. Adipose tissue synthesizes proinflammatory cytokines such as TNF-*α*, interleukins, and cytokine-like proteins known as adipokines [39], and CRP (an acute phase protein) is primarily derived from IL-6 hepatic biosynthesis [40].

This low-grade proinflammatory and prothrombotic state present by T2DM and obesity is an inseparable condition of atherosclerosis and might confer an increased propensity to accelerated atherogenesis and macrovascular complications [23]. Previously, Mita et al. reported an independent association between hs-CRP and subclinical atherosclerosis in early-state T2DM [41]. Also, Schulze Horn et al. reported an independent relationship between hs-CRP and subclinical atherosclerosis in a population-based sample [42]. Despite the lack of clinical manifestations of CVD in our sample, we cannot dismiss the presence of subclinical atherosclerosis.

Biochemical markers, such as hs-CRP and fibrinogen have been used for evaluating the cardiovascular risk and incipient diabetes [18, 43, 44]. Although none of those biomarkers improve diabetes prediction and the magnitude of the risk varies according to the type of CVD, the presence of higher levels of proinflammatory and prothrombotic markers in our study must encourage us for maintenance of an adequate control of the traditional cardiovascular risk factors to prevent the deterioration of atherosclerosis and consequently the development of CVD.

There are strengths in our study. Although this is a cross-sectional nonrandomized assay, the gender and smoking status distribution were similar between all three groups and did not act as cofounder variables. A low grade of obesity assessed by BMI was present in the T2DM group, diminishing the effects of obesity in serum hs-CRP levels. Also, individuals with T2DM were in optimal glucose control assessed by FPG and HbA1c concentrations. Our study had some limitations. Even though all individuals did not have clinical manifestations of CVD at the time of the enrollment, we cannot discharge the influence of subclinical atherosclerosis in the results. Furthermore, some potential cofounders such as physical activity and alcohol intake were not incorporated into the analysis. In addition, cases of IGT assessed by FPG plus HbA1c were limited and could not been evaluated. Also, this research was conducted in one center only. Therefore, more studies are needed to confirm our results.

Numerous publications indicate a significant contribution of inflammatory and procoagulant factors to the development of atherosclerosis and diabetes. Also, inflammatory and procoagulant markers, as well as hyperglycemia, increase the cardiovascular risk. Previous studies had demonstrated that an increased visceral adipose tissue mass is associated with increased C-reactive protein in patients with manifest vascular diseases. Our findings suggest a subclinical proinflammatory and prothrombotic state in individuals with T2DM without clinical manifestations of cardiovascular disease. We consider that those markers could be a useful tool to screen subjects with higher cardiovascular risk, in order to prevent an atherothrombotic disease such as myocardial infarction or stroke.

## 5. Conclusions

Proinflammatory and prothrombotic state are present in people with T2DM lacking clinical history of CVD. Fibrinogen and hs-CRP serum concentrations are correlated and predominantly determined by BMI. The increased concentration of fibrinogen and hs-CRP added to other cardiovascular risk factors in a same individual might accelerate the development and severity of CVD, and preventive measures should be encouraged to improve glycemic control and reduce BMI.

## Figures and Tables

**Table 1:** Demographic and clinical characteristics of the overall sample categorized according to glucose tolerance status.

Total = 939 individuals			
Parameter	NGT *n* = 473 (50.4%)	Pre-DM *n* = 250 (26.6%)	T2DM *n* = 216 (23%)
Age (years)			
Mean ± SD	48.5 ± 12.9	52 ± 10.6*	55.8 ± 10.4^∗†^
Median [IQR]	50 [40–58]	52 [45–59]	57 [49–62]
Women, *n* (%)	328 (69.3)	168 (67.2)	142 (65.7)
BMI (kg/m^2^)			
Mean ± SD	28 ± 4.6	29.3 ± 4.4*	30.4 ± 5.2^∗†^
Median [IQR]	27.5 [24.7–31]	29 [26.1–31.7]	29.6 [26.8–32.8]
BMI ≥ 30 kg/m^2^, *n* (%)	150 (31.7)	99 (39.6)	102 (47.2)*
WC (cm)			
Mean ± SD	91.3 ± 12.2	95.6 ± 9.9*	98.3 ± 11.2^∗†^
Median [IQR]	91 [82–99]	95 [89–102]	98 [91-105]
Hypertension, *n* (%)	137 (28.9)	77 (30.8)	117 (54.1)^∗†^
Currents smoking, *n* (%)	107 (22.6)	48 (19.2)	48 (22.2)
FH of T2DM, *n* (%)	234 (49.4)	146 (58.4)	147 (68)*
FH of CVD, *n* (%)	71 (15)	38 (15.2)	34 (15.7)

Data are means ± standard deviation, median [interquartile range], and percentages; *P value ≤ 0.05 versus NGT; ^†^ *P* value ≤ 0.05 versus pre-DM; NGT: normal glucose tolerance; pre-DM: pre-diabetes mellitus; T2DM: type 2 diabetes mellitus; BMI: body mass index; WC: waist circumference; FH of T2DM: familial history of type 2 diabetes mellitus; FH of CVD: familial history of cardiovascular disease.

**Table 2:** Biochemical characteristics of the overall sample categorized according to glucose tolerance status.

Total = 939 individuals			
Parameter	NGT *n* = 473 (50.4%)	Pre-DM *n* = 250 (26.6%)	T2DM *n* = 216 (23%)
FPG (mg/dL)			
Mean ± SD	89.6 ± 6.4	107 ± 5.9*	158.1 ± 68.1^∗†^
Median [IQR]	90 [86–95]	105 [102–111]	135 [111–181]
HbA1c (%)			
Mean ± SD	4.0 ± 0.5	4.2 ± 0.8*	5.9 ± 2.1^∗†^
Median [IQR]	3.9 [3.7–4.2]	4.1 [3.9–4.5]	5.2 [4.4–7.2]
HDL-C (mg/dL)			
Mean ± SD	40.8 ± 12.7	39 ± 10.3	37.4 ± 10.2*
Median [IQR]	39.2 [31.6–48.5]	37.1 [31.4–46]	35.9 [30.5–42.4]
Triglycerides (mg/dL)	184.1 ± 117.2	222.3 ± 154.8*	231.8 ± 301.2*
Median [IQR]	159 [109–219]	182 [129–266]	191 [127–264]
Fibrinogen (mg/dL)			
Mean ± SD	369.8 ± 69.1	372 ± 69.8	385.9 ± 77.8*
Median [IQR]	365 [322–408]	370 [323–418]	377 [330–438]
hs-CRP (mg/L)			
Mean ± SD	0.4 ± 0.5	0.4 ± 0.7	0.5 ± 0.6*
Median [IQR]	0.3 [0.1–0.4]	0.2 [0.1–0.5]	0.3 [0.1–0.6]

Data are means ± standard deviation, median [interquartile range] and percentages; *P value ≤ 0.05 versus NGT; ^†^ *P *value ≤ 0.05 versus pre-DM; NGT: normal glucose tolerance; preDM: pre-diabetes mellitus; T2DM: type 2 diabetes mellitus; FPG: fasting plasma glucose; HbA1c: glycosylated hemoglobin; HDL-C: high-density lipoprotein colesterol; hs-CRP: high sensitivity C-reactive protein.

**Table 3:** Correlation analysis of proinflammatory and prothrombotic markers with traditional cardiovascular risk factors.

Total = 939 individuals									
	Age	BMI	WC	FPG	HbA1c	HDL-C	Tg	hs-CRP	Fibrinogen
Fibrinogen	**0.14**	**0.25**	**0.18**	**0.10**	**0.14**	0.04	0.005	**0.53**	—
hs-CRP	−0.01	**0.33**	**0.27**	**0.14**	**0.12**	−0.09	0.07	—	**0.53**

Dependent variables are listed vertically, and covariates are listed horizontally. Cells show univariate correlation coefficients (significant in bold *= P* ≤ 0.05). BMI: body mass index; WC: waist circumference; FPG: fasting plasma glucose; HbA1c: glycosylated hemoglobin; HDL-C: high-density lipoprotein cholesterol; Tg: triglycerides; hs-CRP: high sensitivity C-reactive protein.

**Table 4:** Multiple linear stepwise regression analysis for fibrinogen and hs-CRP as dependent variables.

Total = 939 individuals				
Independent variables	Fibrinogen		Hs-CRP	
	**β** (95% CI)	*P* value	*β* (95% CI)	*P* value
Age	0.14 (0.06–0.23)	**0.001**	—	*— *
BMI	0.14 (−0.008–0.29)	*0.06 *	0.19 (0.04–0.34)	**0.010**
WC	−0.08 (–0.23–0.06)	0.24	—	*— *
FPG	−0.10 (−0.23–0.03)	0.14	0.11 (−0.02–0.24)	0.10
HbA1c	0.12 (−0.003–0.25)	**0.05**	−0.06 (−0.19–0.06)	0.32
Fibrinogen	—	—	0.48 (0.39–0.56)	**<0.0001**
Hs-CRP	0.51 (0.42–0.60)	**<0.0001**	—	*— *
*R* ^2^	**0.31**		**0.33**	

Dependent variables are listed horizontally, and independent variables are listed vertically; *β*: standardized correlation coefficients (95% confidence interval); *R* ^2^: adjusted determination coefficient (significant in bold *= P* ≤ 0.05); BMI: body mass index; WC: waist circumference; FPG: fasting plasma glucose; HbA1c: glycosylated hemoglobin; hs-CRP: high sensitivity C-reactive protein.
